# Parents' Perceptions of the Challenges to Helping Their Children Maintain or Achieve a Healthy Weight

**DOI:** 10.1155/2019/9192340

**Published:** 2019-01-09

**Authors:** Sara N. Bleich, Mary T. Gorski Findling, Robert J. Blendon, Eran Ben-Porath, Gillian K. SteelFisher

**Affiliations:** ^1^Health Policy and Management, Harvard Chan School of Public Health, Boston, Massachusetts, USA; ^2^Harvard University, Cambridge, Massachusetts, USA; ^3^Public Opinion Research, SSRS, Glen Mills, PA, USA

## Abstract

**Introduction:**

Parents play a critical role in their children's weight. This paper examines parents' perceptions about the challenges to helping their kids maintain or achieve a healthy weight.

**Methods:**

We analyzed data in 2017 from a U.S. telephone poll conducted during October-November 2012 among parents or caregivers of children aged 2–17 years using a nationally representative sample of households. It included 667 White, 123 Black, and 167 Hispanic parents. Multiple logistic regressions were used to examine parent perceptions about the individual- and environmental-level challenges to helping their children maintain or achieve a healthy weight.

**Results:**

Overall, 45% of children have parents who reported challenges helping the child eat to maintain or achieve a healthy weight, and 35% have parents who reported challenges for exercise. According to parents, most children consumed snacks between 3 pm and bedtime during the school week (83%), and 63% of those children had an unhealthy snack. Parents did not express much concern about unhealthy snacks; 80% of children had parents who said that they did not mind since their child generally ate healthy food. Children with Hispanic and Black parents were more likely than those with White parents to have parents reporting environment challenges, such as unhealthy foods in schools.

**Conclusions:**

Helping children maintain a healthy weight through diet is a problem for many parents, regardless of their race or ethnicity. Differences by race/ethnicity in parent perceptions of food environment challenges to helping their child maintain or achieve a healthy weight suggest possible areas for future interventions.

## 1. Introduction

Childhood obesity is a major public health problem in the United States. It currently affects 1 in 5 children [1] with disproportionate impact among children in minority racial/ethnic groups [2, 3]. It is estimated that more than half of the children today (57%) will be obese at the age of 35 years [4]. Parents play a critical role in their children's weight [5, 6] and can help their children develop and maintain healthy eating and physical activity habits. For example, parental practices and household routines shape their children's overall obesity risk [7], dietary patterns (e.g., unhealthy snacking) [6, 8], and physical activity [9].

While parents uniformly recognize obesity as a serious problem at the national level [10], many struggle with how to effectively encourage healthy behaviors in their children. Parents also consistently overestimate the healthfulness of their children's diets and physical activity levels [11–13] and underestimate their children's weight, often failing to recognize when their child is overweight or obese [14–19]. Those parents who do perceive their children to be overweight or obese are typically more likely to encourage behavioral changes [20].

Given that parents can play an important role in addressing the problem of obesity, a better understanding of their perceived challenges to facilitating healthy weight among their children, particularly among children at higher risk for obesity, may help identify effective future interventions. The objective of this study was to use a national sample of households with children to explore parents' perceptions of eating- and exercise-related challenges to helping their children maintain or achieve a healthy weight, overall and by racial/ethnic groups.

## 2. Methods

### 2.1. Data and Design

Data for this study come from a poll designed by researchers at Harvard T. H. Chan School of Public Health (Boston, MA). The poll was fielded by an independent firm, SSRS (Media, PA), from October 11 to November 21, 2012, using a nationally representative, randomized telephone sample (including both landline and cellular phones) of households with children aged 2–17 years. The data were analyzed in 2017, undertaken with knowledge that childhood obesity rates and children's diet/exercise patterns have remained relatively steady in recent years [21, 22] and were unlikely to have changed significantly since 2012. Data are available on request. A randomly selected child from each sampled household was the focus of questions, and caregivers were screened for being able to answer questions about what that child had done the previous day in order to minimize biases in the estimates of children's activities and consumption. Thus, the sample is representative of children in the United States aged 2–17 years, as described by knowledgeable caregivers. The final sample included 1,018 caregivers. Caregivers were primarily parents (87%), and also grandparents, siblings, aunts, uncles, and foster parents (referred to as “parents” throughout this analysis). Interviews were conducted Tuesday through Saturday so that respondents answered questions about the Monday-Friday work/school week. The margin of error for total households is ±4.1 percentage points at the 95% confidence level.

To compensate for known biases in telephone surveys (e.g., nonresponse bias) and for variations in probability of selection within and across households, sample data are weighted by household size and composition, homeownership, cell phone/landline use, and demographics of the child (gender, age, race/ethnicity, metro status, and census region) to reflect the true population of children aged 2–17 years in the country. Other techniques, including random-digit dialing, replicate subsamples, and systematic respondent selection within households, were used to ensure that the sample is representative.

The completion rate was 63% (64% landline and 63% cell phone). The response rate for this survey was calculated based on the American Association for Public Opinion Research's (AAPOR) RR3 formula. By this calculation, response for the landline component of this study was 22% and for the cell phone was 15%; this is lower than that of many longer-term demographic surveys but is within the typical range of response rates for telephone polling [23]. Because data from this study were drawn from a probability sample and used the best available sampling and weighting practices in polling methods, they are expected to provide accurate results consistent with surveys with higher response rates [24–28]. The data were considered exempt from IRB approval and deidentified.

### 2.2. Measures

The main outcomes for this study were parent perceptions of factors that made it hard for them to help their child maintain or achieve a healthy weight, including (1) overall level of challenges in supporting eating/exercise, (2) specific food and exercise environmental challenges (school and community), and (3) unhealthy snacking behaviors and related reasoning. The survey questions and response categories can be found in the Appendix.

#### 2.2.1. Overall Level of Eating/Exercise Challenges to Achieving a Healthy Weight

Overall eating/exercise challenges were based on survey questions asking parents about difficulty making sure that their child ate and exercised in ways that helped them maintain or achieve a healthy weight. Questions were answered on a 4-point scale (very difficult, somewhat difficult, not very difficult, and not difficult at all), which we dichotomized to difficult (very/somewhat) or not (not very/not at all).

#### 2.2.2. Environmental Challenges to Achieving a Healthy Weight

Environmental challenges were based on a question asking parents to identify whether each factor in a list (e.g., lots of unhealthy advertising, lack of fruits and vegetables in nearby stores, unhealthy stores near the child's school, and lack of safe places to exercise) was a problem (major or minor) or not in helping their child maintain or achieve a healthy weight.

#### 2.2.3. Unhealthy Snacking Behaviors and Related Reasoning

Unhealthy snacking behaviors were based on survey questions asking parents if their child ate or drank between meals (after 3 pm and before dinner or after dinner and before bed) and if the parent knew what the child consumed. Among parents whose children had a snack that they could identify (*n*=810), we created a measure of unhealthy snacking by summing the percentage of parents reporting their child had any unhealthy foods or beverages as snacks. Unhealthy foods were defined as “foods that can lead to unhealthy weight gain” or “foods with a high fat or sugar content, like chips, fried foods, fast foods, or sweets” and unhealthy beverages were defined as “drinks that can lead to unhealthy weight gain” or “sweetened drinks like nondiet soda or sports drinks.” Parents whose child consumed unhealthy snacks (*n*=667) were then given a list and asked to identify whether or not several factors (e.g., lack of time to shop or too tired to make something different) were reasons their child consumed unhealthy snacks.

### 2.3. Statistical Analyses

All analyses were conducted using STATA version 15.0 (StataCorp, College Station, TX) with survey weights to account for the complex sampling design. For each of the outcome variables, multiple logistic regression was used to adjust for potential differences in population characteristics, including the child's age (2–6, 7–11, and 12–17) and gender, parent's age (18–34, 35–49, and 50+) and gender, parent's education (high school or less, some college, and college+), household income (less than $20,000, $20,000-less than $50,000, and $50,000+), household composition (1-parent or 2-parent), and mean number of children in the household. In addition, we controlled for additional factors known to affect obesity-related behaviors, including whether parents or siblings were overweight (yes/no) [29] and parent perception of the child's weight (overweight, about right, and underweight) [19]. Using postestimation commands, we calculated predicted probabilities of each study outcome, adjusting for covariates. We tested overall group differences and examined post hoc pairwise comparisons of differences across the three race/ethnicity categories. For all models, statistical significance was determined at *p* < 0.05. All covariates in Table 1 were included in the models, regardless of statistical significance. Surveyed households with parents who were not non-Hispanic White, non-Hispanic Black, or Hispanic (*n*=61) were dropped from the analysis.

## 3. Results

Characteristics of the sample and parent respondents are presented in Table 1, overall and by parent race/ethnicity. Overall, 68% of children had parents reporting for this survey who were female, 50% had reporting parents who were aged 35 to 49 years, 62% had reporting parents with some college education or more, and 73% had reporting parents who perceived their weight as about right. Overall, among children, 49% were female, 60% were aged 2 to 11 years, and 35% had parents or siblings who were overweight. Overall, 49% of children were in households with an income greater than $50,000, 68% of children had two parents, and the average number of children per household was 2.4. There were significant differences by parent race/ethnicity in parents' age, education, household income, household composition, and perception of their child's weight.

Table 2 reports differences in parents' reporting of the overall level of challenges in supporting eating/exercise and related food/exercise environment challenges in helping their child eat to maintain or achieve a healthy weight, overall and by race/ethnicity. Almost half of the children had parents who reported challenges with helping their children eat to maintain or achieve a healthy weight (45%). With respect to food environmental-level challenges to helping their child eat to maintain or achieve a healthy weight, 44% of children had parents who were most likely to identify lots of advertising for unhealthy foods, followed by unhealthy school lunches or vending (32%). Children with Hispanic parents had a higher probability of parents reporting unhealthy school lunches or vending compared to children with White and Black parents (White: 29%, Black: 24%, and Hispanic: 50%). Children with Hispanic parents were also more likely to have parents reporting that healthy school food was expensive compared to children with Black parents (Black: 17% and Hispanic: 39%). Children with Black or Hispanic parents were more likely than children with White parents to have parents reporting there was unhealthy food available very close to their child's school (White: 17%, Black: 41%, and Hispanic: 38%).

Table 3 shows the differences in the probability of children having parents reporting challenges with their child's snacking behaviors that may lead to unhealthy weight gain, overall and by race/ethnicity. Overall, most children had parents who reported that their child had a snack (food and/or beverage) between 3 pm and bedtime (83%), and 63% of those children had an unhealthy snack. The most common reason parents gave for why their child had an unhealthy snack (food and/or beverage) was that they did not mind since their child generally ate healthy food; 80% of children had parents reporting this. The second most common reason was because the child liked the taste of the snack; 74% of children had parents reporting this. Children with Black parents were more likely to have parents reporting that their children had unhealthy beverages compared to children with White parents (White: 26% vs. Black: 47%). Children with Hispanic parents were more likely than children with White parents to have parents reporting lack of time as a reason for why their child consumed unhealthy snacks (White: 24% vs. Hispanic: 48%). Children with White or Hispanic parents were more likely than children with Black parents to have parents say that it was too expensive to get healthier snacks (White: 13%, Black: 4%, and Hispanic: 21%).

Table 4 reports the differences in parent perceptions related to overall level of challenges in helping their child exercise to maintain or achieve a healthy weight, overall and by race/ethnicity. Overall, one-third of children had parents who reported that it was very or somewhat difficult to make sure their child exercises in a way that helped them maintain or achieve a healthy weight (35%), with no statistically significant differences across parent race/ethnicity. Children with Black parents were more likely to have parents reporting insufficient places for safe exercise nearby compared to children with White parents (White: 16% vs. Black: 42%). Children with Hispanic parents were less likely to have parents citing lack of good sidewalks nearby for exercise as a problem in helping their child to maintain or achieve a healthy weight (White: 37%, Black, 46%, and Hispanic: 12%).

## 4. Discussion

This study provides important insights into parents' perceptions of challenges in making sure their children eat and exercise in ways that allow them to maintain or achieve a healthy weight. Nearly half of the children have parents who—regardless of race or ethnicity—reported daily difficulty helping their child eat in order to maintain or achieve a healthy weight.

Also notable are our findings related to food environment-level problems helping children maintain or achieve a healthy weight. The most commonly reported challenges were the large volume of unhealthy food advertising and unhealthy food at school (lunch or vending machines). Although the level of challenges with children's eating were similar across racial and ethnic groups, parents' perceptions of environmental problems differed by race/ethnicity. Children with Hispanic parents were more likely than children with Black or White parents to have parents reporting problems with school foods. Children with Hispanic or Black parents were more likely than those with White parents to have parents reporting unhealthy food close to school. Children with Black parents had the highest likelihood of parents reporting unhealthy beverage consumption when snacking and environmental problems related to exercise.

Significant changes to the U.S. nutrition policy since this survey was fielded may be helpful to parents who report challenges with unhealthy food and beverages at school. For example, the Healthy, Hunger-Free Kids Act of 2010 [30] aligns the National School Lunch and School Breakfast Programs with the 2010 *Dietary Guidelines for Americans* and requires all foods sold at schools to meet nutrition standards (Smart Snacks) [31]. Specifically, the legislation aims to make the school nutrition environment healthier for children and reduce obesity by improving nutritional standards for school foods and beverages, decreasing barriers to accessing school meals (e.g., Community Eligibility Provision), providing grant funding (e.g., Farm to School and kitchen equipment upgrades), providing training and technical assistance for school food service professionals, and creating new provisions for local school wellness policies. Because this law was only fully implemented beginning in the 2016-2017 school year, it is not yet clear how well these new policies have been operationalized, whether they have helped reduce racial and ethnic disparities in access to healthy and affordable foods or how much they will change children's long-term diet patterns. Emerging evidence suggests that it is likely that despite existing improvements in the school nutrition environment, there is still a need for more science-based policies and practices at the state and local levels to promote healthier food environments, particularly for children at higher risk for obesity [32, 33]. One promising policy alternative to reduce a major source of excess calorie intake in children that is gaining momentum is sugary beverage taxes, which has been passed in eight U.S. localities (Berkeley; Philadelphia; Albany, CA; Boulder; Cook County; Oakland; San Francisco; and Seattle), is being considered in several more localities.

This study builds evidence that parents have an unrealistic view of their children's eating and exercise habits. Our findings are consistent with other literature showing high parental concern over children's weight and obesity [10]. Despite this, parents appear to express minimal concern about their children's unhealthy (energy-dense, nutrient poor) snack consumption—a potential contributor to children's weight status. This may stem from parents consistently overestimating the healthfulness of their children's diets, despite a high prevalence of unhealthy snacking among youth in the United States. Most children's snacking and overall diet patterns do not follow the *Dietary Guidelines for Americans*, and energy-dense, nutrient-poor snacks are widely available in settings where children spend a significant amount of time, including at home, in schools, and in retail stores [11, 13, 34, 35]. This study supports other research works finding that a significant share of parents believe it is acceptable to offer children “treats” every day [36]. Thus, future strategies to improve children's diets should aim to create healthier norms around snacking habits in schools, at home, and away from home, including eliminating children's daily consumption of unhealthy snacks during the school week.

Parental perceptions of physical activity are also concerning, as prior research shows that nationally, the vast majority of children (78%) do not obtain the recommended amount of physical activity each week [37]. Despite this low national prevalence of physical activity, we found that only one-third of the children's parents expressed difficulty in helping their children exercise to maintain or achieve a healthy weight. This lack of expressed difficulty suggests that most parents wrongly consider their children to be sufficiently active when they are inactive, and health professionals should address this gap in future strategies to promote physical activity [12, 38]. We also found that between one-quarter and one-half of children had parents who reported multiple problems with their school food environment, with a higher prevalence of concerns reported by children with Hispanic and Black parents. Prior studies have shown wide public support for school-based interventions to prevent or reduce obesity [39, 40], suggesting that interventions to improve the healthfulness of school foods may address many of the parental concerns documented in this study.

This study is subject to several limitations, including the cross-sectional nature of the data. It is possible that differential nonresponse bias across race/ethnicity may not have been fully addressed through weighting. However, it is likely that the magnitude of differences in nonresponse across race/ethnicity is small, and weighting corrections within groups make differences in nonresponse unlikely. Because this analysis used multiple comparisons, it may have resulted in more statistically significant findings than actually exist in the population, but the racial/ethnic differences reported were large enough that problems from multiple comparisons are unlikely to have played a meaningful role in the study conclusions. In addition, question wording and ordering effects may bias results, but questions in lists were randomized or rotated, and all questions were pretested to ensure respondent understanding ahead of time. This study focused on perceptions during the school week and may not be representative of challenges that parents face during the weekend. These data were collected in 2012, and the challenges parents face may have shifted over time, although children's diet and exercise patterns in the United States have remained relatively steady from 2012 to 2018.

This study also had several strengths, including a nationally representative sample of households with children, and several understudied measures of parents' perceptions of their child's eating and exercise habits. This is also the first study to our knowledge that examined parents' perceptions of the daily challenges and food environment challenges to helping their children maintain or achieve a healthy weight.

## 5. Conclusion

Helping their children achieve or maintain a healthy weight through diet is a problem for many parents, regardless of their race or ethnicity. Parents have an unrealistic view of their children's eating habits. According to parents, most children consume unhealthy snacks during the school week, but parents are unconcerned since they believe their children generally follow a healthy diet. Encouraging health professionals to recognize and address these discrepancies between parent perceptions and their children's eating behavior may help facilitate important behavior change among children. In addition, differences by race/ethnicity in parent perceptions of food environment challenges to helping their child maintain or achieve a healthy weight (such as children with Black or Hispanic parents being more likely to report unhealthy food close to their child's school) suggests possible areas for future interventions. Continued proliferation of policy alternatives to improve the food environment (e.g., school food policies and sugary beverage taxes) is important to help children achieve or maintain a healthy diet.

## Figures and Tables

**Table 1:** Characteristics of the study sample, by parent^+^ race/ethnicity.

	Total	White (non-Hispanic)	Black (non-Hispanic)	Hispanic	*p* value^*∗*^
	*n* (weighted %)	*n* (weighted %)	*n* (weighted %)	*n* (weighted %)	
Total	957	667 (64)	123 (15)	167 (21)	—
Parent gender					0.2247
Male	331 (32)	250 (34)	40 (31)	41 (25)	
Female	626 (68)	417 (66)	83 (69)	126 (75)	
Child gender					
Male	473 (51)	326 (52)	61 (48)	86 (49)	0.7334
Female	483 (49)	340 (48)	62 (52)	81 (51)	
Parent's perception of the child's weight					**0.0017** ^**∗**^
Overweight	142 (15)	83 (11)	29 (29)	30 (17)	
About right	677 (73)	473 (75)	84 (61)	120 (75)	
Underweight	137 (12)	110 (14)	10 (10)	17 (8)	
Parent age					**0.0002** ^**∗**^
18–34 y	220 (31)	127 (25)	31 (38)	62 (43)	
35–49 y	508 (50)	384 (56)	46 (34)	78 (44)	
≥50 y	228 (19)	155 (19)	46 (28)	27 (12)	
Child age					0.9334
2–6 y	262 (30)	189 (31)	30 (28)	43 (28)	
7–11 y	289 (30)	196 (29)	41 (33)	52 (32)	
12–17 y	406 (40)	282 (40)	52 (40)	72 (40)	
Education					**<0.0001** ^**∗**^
High school or less^2^	289 (38)	138 (25)	53 (51)	98 (69)	
Some college^3^	238 (24)	171 (26)	37 (30)	30 (13)	
College+	427 (38)	356 (49)	33 (19)	38 (18)	
Household income					
<$20,000	107 (18)	36 (9)	30 (33)	41 (35)	**<0.0001** ^**∗**^
$20,000–<$50,000	236 (33)	129 (28)	38 (38)	69 (47)	
$50,000+	543 (49)	458 (63)	48 (29)	37 (19)	
Household composition					**<0.0001** ^**∗**^
1-parent	243 (32)	130 (26)	66 (64)	47 (27)	
2-parent	674 (68)	515 (74)	44 (36)	115 (73)	
Number of children in household M ± SD	2.4 ± 0.1	2.3 ± 0.1	2.5 ± 0.2	2.4 ± 0.1	0.6544
Parent or siblings overweight	350 (35)	245 (35)	49 (40)	56 (34)	0.6479

*Note.* Boldface indicates statistical significance (*p* < 0.05). Data are based on a poll that was fielded from October 11 to November 21, 2012, using a nationally representative, randomized telephone sample (including both landline and cellular phones) of households with children aged 2–17 years. ^*∗*^ *p* values for difference is based on the chi-squared test for categorical demographic variables and OLS regression for continuous demographic variables. Significance level *p* < 0.05. ^+^In this survey, the parent is synonymous with nonparent primary caregiver respondents. ^1^Percentage of U.S. population estimated with survey weights to adjust for unequal probability of sampling; ^2^those with a high school degree or GED certificate; ^3^attendance at a business, technical, or vocational school after high school.

**Table 2:** Differences in the probability of reported overall daily challenges in helping their children eat to maintain or achieve a healthy weight, by parent race/ethnicity (*n*=840).

		Parent race/ethnicity		
	Total	White (non-Hispanic)	Black (non-Hispanic)	Hispanic
	Predicted probability (95% CI)	Predicted probability (95% CI)	Predicted probability (95% CI)	Predicted probability (95% CI)
*Daily challenges to help the child achieve a healthy weight*				
Difficulty^1^ in making sure the child eats for a healthy weight	44.7% (40.4, 48.9)	48.2% (42.5, 53.9)	36.5% (24.4, 48.7)	39.5% (29.6, 49.4)
*Problems* ^2^ *with helping the child maintain or achieve a healthy weight* ^3^				
Lots of advertising for unhealthy foods	44.2% (35.3, 49.1)	45.1% (36.4, 53.8)	26.0% (4.9, 47.0)	44.5% (27.6, 61.4)
School lunch/vending is unhealthy	31.6% (26.7, 36.6)	28.9%^c^ (23.1, 34.8)	23.5%^c^ (8.0, 38.9)	50.1%^ab^ (37.3, 62.8)
Nearby stores do not sell reasonably priced fruits and vegetables	26.6% (20.4, 32.8)	28.3% (20.2, 36.4)	27.2% (6.1, 48.3)	22.1% (9.2, 35.1)
Few social venues (restaurants or malls) serving healthy food	29.7% (24.0, 35.4)	27.1% (20.3, 33.9)	41.8% (22.1, 61.4)	28.2% (12.9, 43.4)
Healthy school food is expensive	26.2% (19.9, 32.6)	26.8% (17.9, 35.7)	16.6%^c^ (2.9, 30.4)	38.5%^b^ (22.1, 54.8)
Unhealthy food very close to school	25.4% (19.4, 31.5)	17.4%^bc^ (11.3, 23.5)	40.6%^a^ (20.4, 60.7)	37.5%^a^ (20.6, 54.4)

*Note.* All Table 2 estimates adjust for parent gender, child gender, parent age, child age, parent education, household composition (1- or 2-parent), household income, parent perception of child's weight, number of children in household, and whether parents or siblings are overweight. ^a^Significantly different from non-Hispanic Whites at *p* < 0.05; ^b^significantly different from non-Hispanic Blacks at *p* < 0.05; ^c^significantly different from Hispanics at *p* < 0.05. ^1^Difficult = very/somewhat; ^2^problems = major or minor; ^3^individual questions only asked among a randomized subsample of parents. Data are based on a poll was fielded from October 11 to November 21, 2012, using a nationally representative, randomized telephone sample (including both landline and cellular phones) of households with children aged 2–17 years.

**Table 3:** Differences in the probability of reported child snacking^1^ behaviors, by parent race/ethnicity (*n*=810).

		Parent race/ethnicity		
	Total	White (non-Hispanic)	Black (non-Hispanic)	Hispanic
	Predicted probability (95% CI)	Predicted probability (95% CI)	Predicted probability (95% CI)	Predicted probability (95% CI)
*Child overall snacking*				
Child had a snack (food or beverage) between 3 pm and bedtime	82.6% (79.4, 85.9)	83.1% (79.3, 86.9)	82.8% (72.6, 93.1)	80.5% (71.3, 89.7)
*Unhealthy snacking among children who have had snacks* ^2^				
Child had any *unhealthy* snacks (food or beverage)	63.1% (58.3, 67.8)	60.6% (54.1, 67.1)	74.1% (60.8, 87.4)	63.5% (52.0, 74.9)
Child had any *unhealthy* food	54.2% (49.2, 59.2)	53.1% (46.5, 59.7)	58.6% (44.4, 72.8)	54.6% (42.9, 66.2)
Child had any *unhealthy* beverage(s)	30.1% (25.8, 34.4)	25.6%^b^ (19.8, 31.4)	47.0%^a^ (32.6, 61.3)	30.5% (21.0, 39.9)
*Reasons the child had food/drink that can lead to unhealthy weight gain among children who have had unhealthy snacks* ^3^				
Do not mind since the child generally eats healthy food	80.0% (75.1, 84.8)	85.8% (78.9, 92.8)	74.9% (63.0, 86.8)	72.6% (61.2, 84.0)
Taste of food	73.5% (68.4, 78.5)	73.2% (66.4, 80.1)	77.8% (67.5, 88.1)	69.3% (57.1, 81.5)
Lack of time	32.2% (27.0, 37.4)	24.2%^c^ (17.3, 31.2)	38.7% (24.8, 52.5)	48.2%^a^ (33.4, 63.1)
Too expensive	12.8% (9.3, 16.3)	12.9%^b^ (6.4, 19.4)	3.8%^ac^ (0.0, 8.0)	20.6%^b^ (13.0, 28.2)
Parent was too tired	6.6% (3.9, 9.4)	5.4% (2.2, 8.5)	5.8% (1.2, 10.3)	13.0% (4.4, 21.6)
No adults watching what the child ate	8.9% (5.6, 12.1)	7.4% (3.0, 11.9)	12.6% (1.6, 23.6)	8.8% (2.0, 15.6)

*Note.* All Table 3 estimates adjust for parent gender, child gender, parent age, child age, parent education, household composition (1- or 2-parent), household income, parent perception of child's weight, number of children in household, and whether parents or siblings are overweight; N's differ based on “Don't Know/Refused” responses, variables with missing responses were excluded from models. ^a^Significantly different from non-Hispanic Whites at *p* < 0.05; ^b^significantly different from non-Hispanic Blacks at *p* < 0.05; ^c^significantly different from Hispanics at *p* < 0.05. ^1^Snacking is defined as the parent reported that the child had any food/drink between 3 pm and bedtime yesterday, not including dinner, and parent reported knowing what the child ate/drank; ^2^percent calculated only among the subset of parents reporting their child ate or drank any snacks between 3 pm and bedtime; unhealthy snacks indicate that the parent reported the child had any food/drink that can lead to unhealthy weight gain during this time; ^3^percent calculated only among the subset of parents reporting their child ate or drank any snacks between 3 pm and bedtime that could lead to unhealthy weight gain. Data are based on a poll that was fielded from October 11 to November 21, 2012, using a nationally representative, randomized telephone sample (including both landline and cellular phones) of households with children aged 2–17 years.

**Table 4:** Differences in the probability of reported daily challenges in helping their children exercise to maintain or achieve a healthy weight, by parent race/ethnicity (*n*=838).

		Parent race/ethnicity		
	Total	White (non-Hispanic)	Black (non-Hispanic)	Hispanic
	Predicted probability (95% CI)	Predicted probability (95% CI)	Predicted probability (95% CI)	Predicted probability (95% CI)
*Daily challenges to help the child maintain or achieve a healthy weight*				
Difficulty with making sure the child exercises	34.9% (30.9, 39.0)	34.2% (28.9, 39.4)	29.8% (19.4, 40.1)	41.6% (30.9, 52.3)
*Problems* ^1^ *with helping the child achieve a healthy weight* ^2^				
Insufficient safe places for exercise nearby	22.3% (17.9, 26.7)	15.6%^b^ (11.0, 20.2)	41.9%^a^ (25.8, 57.9)	27.6% (15.8, 39.4)
Lack of good sidewalks, so drive and not walk	33.6% (27.0, 40.2)	36.8%^c^ (29.2, 44.5)	45.9%^c^ (21.7, 70.2)	11.6%^ab^ (2.6, 20.7)
No stores within walking distance	32.2% (25.6, 38.8)	30.9% (21.4, 40.3)	27.9% (10.2, 45.5)	40.0% (23.1, 56.9)
Cost of exercise equipment/gyms	30.8% (24.5, 37.2)	29.9% (21.4, 38.5)	23.9% (11.0, 36.9)	37.3% (22.4, 52.2)

*Note.* All Table 4 estimates adjust for parent gender, child gender, parent age, child age, parent education, household composition (1- or 2-parent), household income, parent perception of child's weight, number of children in household, and whether parents or siblings are overweight. ^a^Significantly different from non-Hispanic Whites at *p* < 0.05; ^b^significantly different from non-Hispanic Blacks at *p* < 0.05; ^c^significantly different from Hispanics at *p* < 0.05. ^1^Problems = major or minor; ^2^questions only asked among a subsample of parents. Data are based on a poll that was fielded from October 11 to November 21, 2012, using a nationally representative, randomized telephone sample (including both landline and cellular phones) of households with children aged 2–17 years.

## Data Availability

The data used to support the findings of this study are available from the corresponding author on request.

## Supplementary Materials

Supplementary Materials The Appendix includes survey questions and response categories used in this analysis.
