# Supplementary material for: Parents' Perceptions of the Challenges to Helping Their Children Maintain or Achieve a Healthy Weight
Source: J Obes. 2019 Jan 9;2019:9192340. doi: 10.1155/2019/9192340 (PMC6343153; doi:10.1155/2019/9192340)
Supplement: Supplementary Materials — The Appendix includes survey questions and response categories used in this analysis. [file 9192340.f1.docx]

**Appendix**

***Daily eating/exercise challenges to achieving a healthy weight***

1. In general, how difficult is it for you to make sure [TCHILD] eats in a way that helps (her/him/them) maintain or achieve a healthy weight? Would you say very, somewhat, not very, or not at all difficult?
2. In general, how difficult is it for you to make sure [TCHILD] exercises in a way that helps (her/him/them) achieve or maintain a healthy weight? Would you say very, somewhat, not very, or not at all difficult?

***Snacking patterns***

For these first questions, please think about the time after 3pm and before dinner [yesterday] specifically.

3. Did [TCHILD] eat or drink anything after 3pm and before dinner yesterday, or didn’t (she/he/they)?

3a. Do you happen to know what (she/he/they) ate or drank during this time, or don’t you?

4. I want to ask about foods that can lead to unhealthy weight gain. By that I mean foods with a high fat or sugar content, like chips, fried foods, fast foods or sweets. How much of that kind of food did (TCHILDNAME) eat yesterday after 3pm and before dinner? Would you say a large amount, a moderate amount, a small amount, or none?

5. Now I’d like to ask about drinks that can lead to unhealthy weight gain. By that I mean sweetened drinks like non-diet soda or sports drinks. How much of those kinds of drinks did (TCHILDNAME) have yesterday after 3pm and before dinner? Would you say a large amount, a moderate amount, a small amount, or none?

*(INTERVIEWER IF ASKED: By sugar sweetened drinks, I mean drinks like iced tea, regular soda like Coca-Cola, sweetened fruit drinks like Kool Aid and Hi-C, and sports or energy drinks like Gatorade or Red Bull. I am not including diet soda or 100% fruit juice in this definition)*

For these next questions, please think about the time after dinner and before (he/she/they) went to bed [yesterday].

6. Did [TCHILD] eat anything after dinner and before (he/she/they) went to bed yesterday, or didn’t (he/she/they)?

6a. Do you happen to know what [TCHILD] ate during this time, or don’t you?

7. I want to ask about foods that can lead to unhealthy weight gain. By that I mean foods with a high fat or sugar content, like chips, fried foods, fast foods or sweets. How much of that kind of food did (TCHILDNAME) eat yesterday after dinner and before going to bed? Would you say a large amount, a moderate amount, a small amount, or none?

8. Now I’d like to ask about drinks that can lead to unhealthy weight gain. By that I mean sweetened drinks like non-diet soda or sports drinks. How much of those kinds of drinks did (TCHILDNAME) have yesterday after dinner and before going to bed? Would you say a large amount, a moderate amount, a small amount, or none?

*(INTERVIEWER IF ASKED: By sugar sweetened drinks, I mean drinks like iced tea, regular soda like Coca-Cola, sweetened fruit drinks like Kool Aid and Hi-C, and sports or energy drinks like Gatorade or Red Bull. I am not including diet soda or 100% fruit juice in this definition)*

9.* You mentioned earlier that (TCHILDNAME) ate at least some food, or had something to drink, that can lead to unhealthy weight gain between 3 PM and the time (he/she/they) went to bed yesterday. For each of the following, please tell me whether or not it was a reason that affected that. What about (INSERT) – was this a reason, or wasn’t it?

1. I was too tired yesterday to get or make something different
2. We were going to different places, and it was easier to get something on the run
3. We haven’t had time to shop, so this was the food we had in the house
4. It’s too expensive to get foods that don’t generally lead to unhealthy weight gain
5. I did not have enough time yesterday to get or prepare something different
6. (TCHILDNAME) likes the taste of this food
7. There were no adults who were watching what (TCHILDNAME) ate
8. As long as my child generally eats healthy foods, I don’t mind if (he/she/they) has these foods sometimes

*For question 9, responses B, C, E coded as *lack of time*.

***Environmental challenges to achieving a healthy weight***

10.* For each of the following, please tell me whether or not it is a major problem, minor problem or not a problem at all in helping (TCHILD) maintain or achieve a healthy weight. How about (INSERT). Would you say this is a major, minor, not a problem, or I am not trying to help my child maintain or achieve a healthy weight?

- 1. The stores nearby do not sell enough reasonably priced fruits and vegetables
  2. (TCHILDNAME) sees a lot of advertising for foods that generally lead to unhealthy weight gain
  3. There aren’t enough places nearby for (TCHILDNAME) to get exercise outside that are safe from traffic
  4. There aren’t enough places nearby for (TCHILDNAME) to get exercise outside where I don’t need to worry about crime
  5. There aren’t good sidewalks near where we live so we often drive instead of walk
  6. There are no stores or other places (TCHILDNAME) might want to go within walking distance so we drive to places farther away
  7. The cost of exercise equipment, gym memberships or team fees for (TCHILDNAME) is too high
  8. At lunchtime, (TCHILDNAME’s) school offers a lot of foods that can lead to unhealthy weight gain
  9. The foods that are low in sugar or fat that (TCHILDNAME’s) school offers for lunch are expensive
  10. The vending machines in (TCHILDNAME’s) school have foods that can lead to unhealthy weight gain
  11. There are places to buy foods that can lead to unhealthy weight gain very close to (TCHILDNAME’s) school
  12. There are few places where (TCHILDNAME) can spend time with friends that are not restaurants or malls serving foods that can lead to unhealthy weight gain

*For question 10, responses C, D coded as *insufficient safe places for exercise nearby*, and responses H & J coded as *school lunch/vending is unhealthy.*
